# Supplementary material for: Guts Bacterial Communities of Porcellio dilatatus: Symbionts Predominance, Functional Significance and Putative Biotechnological Potential
Source: Microorganisms. 2022 Nov 11;10(11):2230. doi: 10.3390/microorganisms10112230 (PMC9692603; doi:10.3390/microorganisms10112230)
Supplement: Supplementary file 1 [file microorganisms-10-02230-s001.zip › Suplementar Figure S4_vfinal_20221103.pptx]

## Slide 1
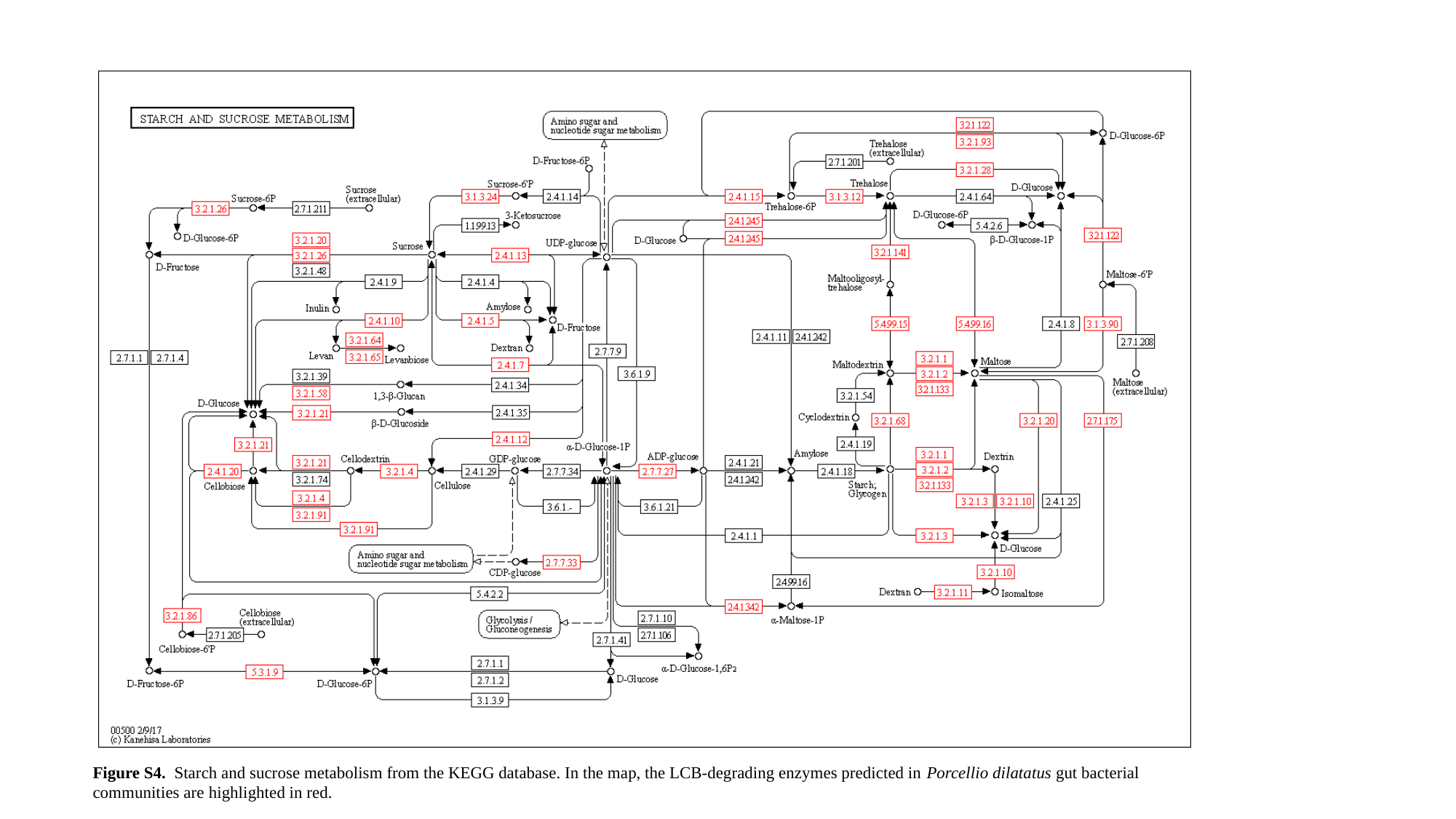

Figure S4. Starch and sucrose metabolism from the KEGG database. In the map, the LCB-degrading enzymes predicted in Porcellio dilatatus gut bacterial communities are highlighted in red.
