# Supplementary material for: Guts Bacterial Communities of Porcellio dilatatus: Symbionts Predominance, Functional Significance and Putative Biotechnological Potential
Source: Microorganisms. 2022 Nov 11;10(11):2230. doi: 10.3390/microorganisms10112230 (PMC9692603; doi:10.3390/microorganisms10112230)
Supplement: Supplementary file 1 [file microorganisms-10-02230-s001.zip › Suplementar Figures1-3_vfinal20221103.pptx]

## Slide 1
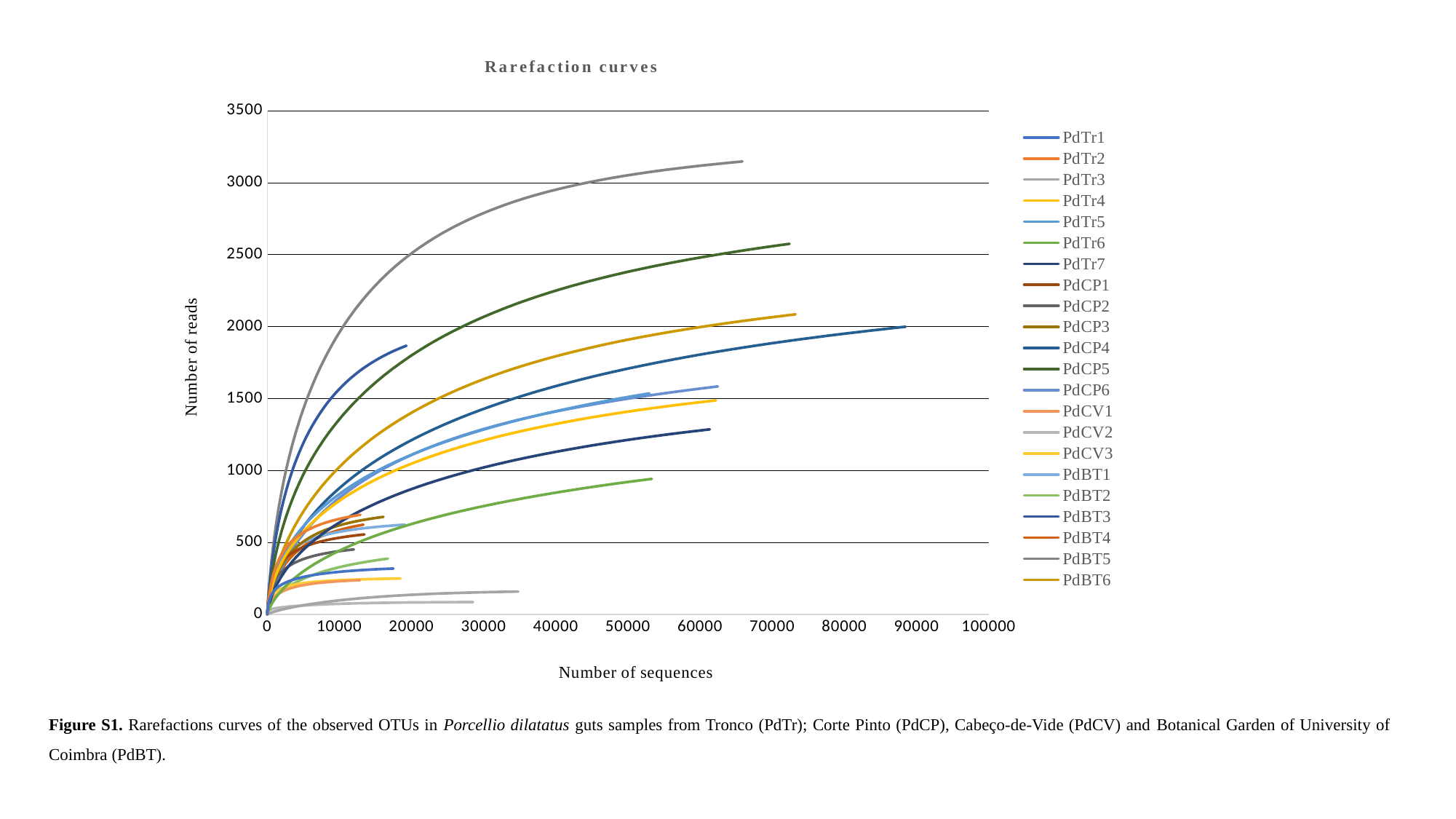

### Chart: Rarefaction curves
| Category | PdTr1 | PdTr2 | PdTr3 | PdTr4 | PdTr5 | PdTr6 | PdTr7 | PdCP1 | PdCP2 | PdCP3 | PdCP4 | PdCP5 | PdCP6 | PdCV1 | PdCV2 | PdCV3 | PdBT1 | PdBT2 | PdBT3 | PdBT4 | PdBT5 | PdBT6 |
|---|---|---|---|---|---|---|---|---|---|---|---|---|---|---|---|---|---|---|---|---|---|---|Figure S1. Rarefactions curves of the observed OTUs in Porcellio dilatatus guts samples from Tronco (PdTr); Corte Pinto (PdCP), Cabeço-de-Vide (PdCV) and Botanical Garden of University of Coimbra (PdBT).

## Slide 2
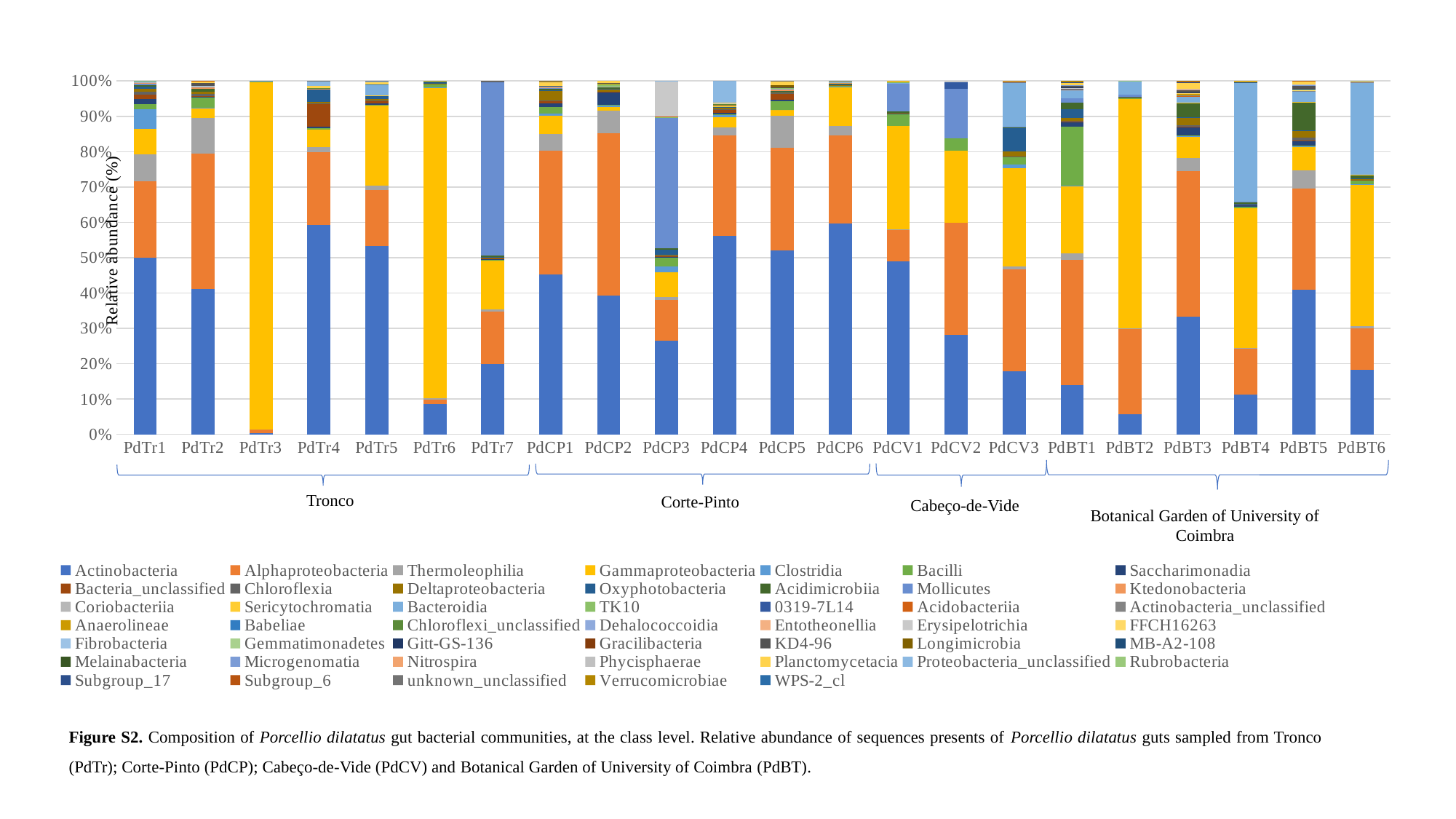

### Chart
| Category | Actinobacteria | Alphaproteobacteria | Thermoleophilia | Gammaproteobacteria | Clostridia | Bacilli | Saccharimonadia | Bacteria_unclassified | Chloroflexia | Deltaproteobacteria | Oxyphotobacteria | Acidimicrobiia | Mollicutes | Ktedonobacteria | Coriobacteriia | Sericytochromatia | Bacteroidia | TK10 | 0319-7L14 | Acidobacteriia | Actinobacteria_unclassified | Anaerolineae | Babeliae | Chloroflexi_unclassified | Dehalococcoidia | Entotheonellia | Erysipelotrichia | FFCH16263 | Fibrobacteria | Gemmatimonadetes | Gitt-GS-136 | Gracilibacteria | KD4-96 | Longimicrobia | MB-A2-108 | Melainabacteria | Microgenomatia | Nitrospira | Phycisphaerae | Planctomycetacia | Proteobacteria_unclassified | Rubrobacteria | Subgroup_17 | Subgroup_6 | unknown_unclassified | Verrucomicrobiae | WPS-2_cl |
|---|---|---|---|---|---|---|---|---|---|---|---|---|---|---|---|---|---|---|---|---|---|---|---|---|---|---|---|---|---|---|---|---|---|---|---|---|---|---|---|---|---|---|---|---|---|---|---|
| PdTr1 | 8543.0 | 3716.0 | 1285.0 | 1231.0 | 954.0 | 262.0 | 221.0 | 220.0 | 165.0 | 127.0 | 119.0 | 73.0 | 71.0 | 36.0 | 25.0 | 25.0 | 18.0 | 13.0 | 0.0 | 0.0 | 0.0 | 0.0 | 0.0 | 0.0 | 0.0 | 0.0 | 0.0 | 0.0 | 0.0 | 0.0 | 0.0 | 0.0 | 0.0 | 0.0 | 0.0 | 0.0 | 0.0 | 0.0 | 0.0 | 0.0 | 0.0 | 0.0 | 0.0 | 0.0 | 0.0 | 0.0 | 0.0 |
| PdTr2 | 4741.0 | 4414.0 | 1175.0 | 313.0 | 19.0 | 316.0 | 45.0 | 19.0 | 60.0 | 85.0 | 0.0 | 81.0 | 0.0 | 52.0 | 0.0 | 0.0 | 42.0 | 1.0 | 0.0 | 0.0 | 0.0 | 0.0 | 0.0 | 2.0 | 0.0 | 0.0 | 2.0 | 0.0 | 0.0 | 0.0 | 0.0 | 0.0 | 88.0 | 0.0 | 0.0 | 0.0 | 0.0 | 0.0 | 0.0 | 63.0 | 0.0 | 0.0 | 0.0 | 12.0 | 0.0 | 0.0 | 0.0 |
| PdTr3 | 134.0 | 284.0 | 1.0 | 33175.0 | 11.0 | 6.0 | 0.0 | 0.0 | 3.0 | 0.0 | 0.0 | 0.0 | 22.0 | 0.0 | 0.0 | 0.0 | 2.0 | 0.0 | 0.0 | 0.0 | 0.0 | 0.0 | 0.0 | 0.0 | 0.0 | 0.0 | 1.0 | 0.0 | 0.0 | 0.0 | 0.0 | 0.0 | 0.0 | 0.0 | 0.0 | 0.0 | 0.0 | 0.0 | 0.0 | 0.0 | 0.0 | 0.0 | 0.0 | 0.0 | 0.0 | 0.0 | 0.0 |
| PdTr4 | 27491.0 | 9520.0 | 629.0 | 2330.0 | 2.0 | 144.0 | 221.0 | 2983.0 | 88.0 | 204.0 | 1649.0 | 66.0 | 0.0 | 6.0 | 0.0 | 0.0 | 63.0 | 16.0 | 0.0 | 0.0 | 0.0 | 0.0 | 17.0 | 4.0 | 0.0 | 0.0 | 0.0 | 0.0 | 0.0 | 0.0 | 0.0 | 0.0 | 1.0 | 1.0 | 0.0 | 0.0 | 0.0 | 0.0 | 0.0 | 250.0 | 539.0 | 0.0 | 0.0 | 0.0 | 113.0 | 0.0 | 0.0 |
| PdTr5 | 25174.0 | 7446.0 | 604.0 | 10648.0 | 1.0 | 149.0 | 170.0 | 376.0 | 47.0 | 111.0 | 489.0 | 8.0 | 2.0 | 1.0 | 0.0 | 17.0 | 1370.0 | 0.0 | 0.0 | 0.0 | 0.0 | 0.0 | 77.0 | 0.0 | 0.0 | 0.0 | 0.0 | 0.0 | 0.0 | 0.0 | 0.0 | 0.0 | 0.0 | 0.0 | 0.0 | 0.0 | 0.0 | 0.0 | 0.0 | 342.0 | 132.0 | 2.0 | 0.0 | 0.0 | 20.0 | 0.0 | 0.0 |
| PdTr6 | 4441.0 | 741.0 | 127.0 | 45914.0 | 149.0 | 506.0 | 17.0 | 73.0 | 4.0 | 4.0 | 263.0 | 9.0 | 0.0 | 1.0 | 58.0 | 0.0 | 2.0 | 2.0 | 0.0 | 0.0 | 0.0 | 0.0 | 1.0 | 0.0 | 0.0 | 0.0 | 1.0 | 0.0 | 0.0 | 0.0 | 0.0 | 0.0 | 0.0 | 0.0 | 0.0 | 0.0 | 0.0 | 0.0 | 0.0 | 17.0 | 3.0 | 2.0 | 0.0 | 0.0 | 0.0 | 0.0 | 0.0 |
| PdTr7 | 11724.0 | 8792.0 | 417.0 | 8107.0 | 5.0 | 140.0 | 86.0 | 212.0 | 23.0 | 16.0 | 188.0 | 131.0 | 29063.0 | 41.0 | 0.0 | 0.0 | 80.0 | 2.0 | 0.0 | 2.0 | 0.0 | 0.0 | 0.0 | 1.0 | 0.0 | 0.0 | 0.0 | 0.0 | 0.0 | 3.0 | 0.0 | 0.0 | 6.0 | 0.0 | 0.0 | 0.0 | 0.0 | 0.0 | 0.0 | 25.0 | 22.0 | 1.0 | 0.0 | 0.0 | 11.0 | 0.0 | 0.0 |
| PdCP1 | 5589.0 | 4314.0 | 592.0 | 638.0 | 61.0 | 239.0 | 121.0 | 83.0 | 15.0 | 355.0 | 13.0 | 54.0 | 31.0 | 8.0 | 0.0 | 16.0 | 3.0 | 0.0 | 0.0 | 0.0 | 0.0 | 0.0 | 0.0 | 0.0 | 0.0 | 0.0 | 0.0 | 0.0 | 0.0 | 22.0 | 0.0 | 0.0 | 9.0 | 0.0 | 0.0 | 0.0 | 0.0 | 0.0 | 0.0 | 129.0 | 0.0 | 0.0 | 0.0 | 0.0 | 29.0 | 19.0 | 0.0 |
| PdCP2 | 4507.0 | 5290.0 | 735.0 | 116.0 | 64.0 | 18.0 | 396.0 | 22.0 | 0.0 | 81.0 | 3.0 | 47.0 | 0.0 | 0.0 | 33.0 | 0.0 | 0.0 | 79.0 | 0.0 | 5.0 | 0.0 | 12.0 | 0.0 | 0.0 | 0.0 | 0.0 | 0.0 | 0.0 | 0.0 | 0.0 | 0.0 | 0.0 | 10.0 | 0.0 | 0.0 | 0.0 | 0.0 | 0.0 | 0.0 | 77.0 | 0.0 | 0.0 | 0.0 | 0.0 | 0.0 | 0.0 | 0.0 |
| PdCP3 | 3981.0 | 1743.0 | 132.0 | 1052.0 | 244.0 | 416.0 | 7.0 | 11.0 | 27.0 | 53.0 | 196.0 | 69.0 | 5583.0 | 8.0 | 0.0 | 0.0 | 2.0 | 14.0 | 0.0 | 8.0 | 0.0 | 0.0 | 0.0 | 0.0 | 0.0 | 0.0 | 1493.0 | 0.0 | 0.0 | 0.0 | 0.0 | 0.0 | 0.0 | 0.0 | 0.0 | 0.0 | 0.0 | 0.0 | 0.0 | 30.0 | 3.0 | 0.0 | 0.0 | 0.0 | 0.0 | 0.0 | 0.0 |
| PdCP4 | 33058.0 | 16661.0 | 1344.0 | 1765.0 | 335.0 | 130.0 | 173.0 | 672.0 | 16.0 | 202.0 | 171.0 | 96.0 | 0.0 | 26.0 | 0.0 | 2.0 | 33.0 | 6.0 | 0.0 | 241.0 | 0.0 | 0.0 | 0.0 | 7.0 | 0.0 | 0.0 | 143.0 | 0.0 | 0.0 | 0.0 | 0.0 | 0.0 | 0.0 | 72.0 | 0.0 | 0.0 | 0.0 | 0.0 | 0.0 | 93.0 | 3581.0 | 0.0 | 0.0 | 0.0 | 3.0 | 0.0 | 0.0 |
| PdCP5 | 28516.0 | 15896.0 | 4920.0 | 880.0 | 4.0 | 1375.0 | 289.0 | 877.0 | 118.0 | 81.0 | 78.0 | 195.0 | 2.0 | 266.0 | 0.0 | 0.0 | 19.0 | 170.0 | 0.0 | 82.0 | 0.0 | 1.0 | 6.0 | 25.0 | 0.0 | 0.0 | 0.0 | 0.0 | 0.0 | 0.0 | 0.0 | 0.0 | 4.0 | 318.0 | 0.0 | 0.0 | 0.0 | 0.0 | 0.0 | 485.0 | 113.0 | 1.0 | 2.0 | 0.0 | 30.0 | 0.0 | 0.0 |
| PdCP6 | 35530.0 | 14834.0 | 1610.0 | 6597.0 | 16.0 | 91.0 | 106.0 | 35.0 | 11.0 | 53.0 | 24.0 | 176.0 | 75.0 | 8.0 | 0.0 | 2.0 | 235.0 | 7.0 | 0.0 | 10.0 | 0.0 | 0.0 | 0.0 | 3.0 | 0.0 | 0.0 | 0.0 | 0.0 | 0.0 | 6.0 | 4.0 | 0.0 | 15.0 | 11.0 | 9.0 | 0.0 | 0.0 | 0.0 | 0.0 | 44.0 | 9.0 | 0.0 | 0.0 | 0.0 | 1.0 | 0.0 | 34.0 |
| PdCV1 | 5957.0 | 1083.0 | 43.0 | 3553.0 | 0.0 | 395.0 | 40.0 | 0.0 | 0.0 | 17.0 | 0.0 | 61.0 | 958.0 | 0.0 | 0.0 | 0.0 | 13.0 | 28.0 | 0.0 | 0.0 | 0.0 | 26.0 | 0.0 | 0.0 | 0.0 | 0.0 | 0.0 | 0.0 | 0.0 | 0.0 | 0.0 | 0.0 | 14.0 | 0.0 | 0.0 | 0.0 | 0.0 | 0.0 | 0.0 | 3.0 | 0.0 | 0.0 | 0.0 | 0.0 | 0.0 | 0.0 | 0.0 |
| PdCV2 | 7987.0 | 9012.0 | 0.0 | 5778.0 | 0.0 | 1024.0 | 0.0 | 0.0 | 0.0 | 0.0 | 0.0 | 0.0 | 3953.0 | 0.0 | 0.0 | 0.0 | 0.0 | 0.0 | 545.0 | 0.0 | 0.0 | 0.0 | 0.0 | 0.0 | 0.0 | 0.0 | 98.0 | 0.0 | 0.0 | 0.0 | 0.0 | 0.0 | 0.0 | 0.0 | 0.0 | 0.0 | 0.0 | 0.0 | 0.0 | 0.0 | 0.0 | 0.0 | 0.0 | 0.0 | 0.0 | 0.0 | 0.0 |
| PdCV3 | 2995.0 | 4823.0 | 138.0 | 4637.0 | 187.0 | 347.0 | 0.0 | 0.0 | 18.0 | 231.0 | 1143.0 | 25.0 | 12.0 | 0.0 | 0.0 | 0.0 | 2074.0 | 0.0 | 0.0 | 0.0 | 41.0 | 0.0 | 0.0 | 0.0 | 0.0 | 0.0 | 13.0 | 0.0 | 0.0 | 0.0 | 0.0 | 25.0 | 0.0 | 0.0 | 0.0 | 0.0 | 0.0 | 0.0 | 0.0 | 18.0 | 0.0 | 0.0 | 0.0 | 0.0 | 0.0 | 0.0 | 0.0 |
| PdBT1 | 2539.0 | 6390.0 | 345.0 | 3434.0 | 31.0 | 3039.0 | 203.0 | 79.0 | 11.0 | 145.0 | 468.0 | 339.0 | 190.0 | 0.0 | 0.0 | 0.0 | 433.0 | 33.0 | 0.0 | 5.0 | 18.0 | 0.0 | 23.0 | 0.0 | 20.0 | 0.0 | 16.0 | 0.0 | 0.0 | 0.0 | 30.0 | 0.0 | 70.0 | 0.0 | 24.0 | 0.0 | 27.0 | 0.0 | 0.0 | 134.0 | 0.0 | 7.0 | 15.0 | 0.0 | 0.0 | 52.0 | 0.0 |
| PdBT2 | 923.0 | 3954.0 | 7.0 | 10595.0 | 0.0 | 24.0 | 47.0 | 4.0 | 0.0 | 2.0 | 1.0 | 1.0 | 124.0 | 0.0 | 0.0 | 0.0 | 615.0 | 0.0 | 0.0 | 0.0 | 0.0 | 0.0 | 0.0 | 0.0 | 0.0 | 0.0 | 0.0 | 0.0 | 0.0 | 0.0 | 0.0 | 0.0 | 4.0 | 0.0 | 0.0 | 0.0 | 0.0 | 0.0 | 0.0 | 2.0 | 0.0 | 3.0 | 0.0 | 0.0 | 0.0 | 0.0 | 0.0 |
| PdBT3 | 5508.0 | 6795.0 | 619.0 | 1004.0 | 15.0 | 50.0 | 394.0 | 6.0 | 64.0 | 381.0 | 15.0 | 638.0 | 13.0 | 0.0 | 0.0 | 2.0 | 320.0 | 3.0 | 0.0 | 11.0 | 27.0 | 75.0 | 10.0 | 0.0 | 0.0 | 0.0 | 0.0 | 16.0 | 29.0 | 0.0 | 2.0 | 0.0 | 108.0 | 0.0 | 11.0 | 0.0 | 0.0 | 10.0 | 43.0 | 284.0 | 0.0 | 19.0 | 6.0 | 18.0 | 0.0 | 39.0 | 0.0 |
| PdBT4 | 1450.0 | 1646.0 | 42.0 | 5061.0 | 0.0 | 52.0 | 59.0 | 10.0 | 6.0 | 30.0 | 2.0 | 82.0 | 4.0 | 0.0 | 0.0 | 0.0 | 4304.0 | 0.0 | 0.0 | 0.0 | 5.0 | 0.0 | 0.0 | 0.0 | 0.0 | 0.0 | 0.0 | 0.0 | 0.0 | 0.0 | 6.0 | 0.0 | 1.0 | 0.0 | 3.0 | 0.0 | 0.0 | 0.0 | 0.0 | 22.0 | 0.0 | 0.0 | 0.0 | 0.0 | 0.0 | 26.0 | 0.0 |
| PdBT5 | 23698.0 | 16489.0 | 3098.0 | 3786.0 | 32.0 | 181.0 | 789.0 | 41.0 | 533.0 | 1022.0 | 62.0 | 4638.0 | 19.0 | 0.0 | 0.0 | 4.0 | 1762.0 | 24.0 | 0.0 | 4.0 | 56.0 | 74.0 | 17.0 | 0.0 | 0.0 | 26.0 | 0.0 | 39.0 | 40.0 | 14.0 | 74.0 | 0.0 | 549.0 | 0.0 | 145.0 | 12.0 | 3.0 | 32.0 | 70.0 | 416.0 | 0.0 | 82.0 | 10.0 | 28.0 | 0.0 | 0.0 | 0.0 |
| PdBT6 | 12806.0 | 8276.0 | 509.0 | 28210.0 | 2.0 | 756.0 | 168.0 | 37.0 | 78.0 | 86.0 | 53.0 | 785.0 | 41.0 | 0.0 | 0.0 | 8.0 | 18400.0 | 2.0 | 0.0 | 0.0 | 19.0 | 8.0 | 0.0 | 0.0 | 0.0 | 1.0 | 0.0 | 12.0 | 7.0 | 0.0 | 4.0 | 0.0 | 47.0 | 0.0 | 14.0 | 0.0 | 0.0 | 8.0 | 3.0 | 71.0 | 0.0 | 25.0 | 0.0 | 0.0 | 0.0 | 0.0 | 0.0 |
Tronco
Corte-Pinto
Cabeço-de-Vide
Botanical Garden of University of Coimbra
Figure S2. Composition of Porcellio dilatatus gut bacterial communities, at the class level. Relative abundance of sequences presents of Porcellio dilatatus guts sampled from Tronco (PdTr); Corte-Pinto (PdCP); Cabeço-de-Vide (PdCV) and Botanical Garden of University of Coimbra (PdBT).

## Slide 3
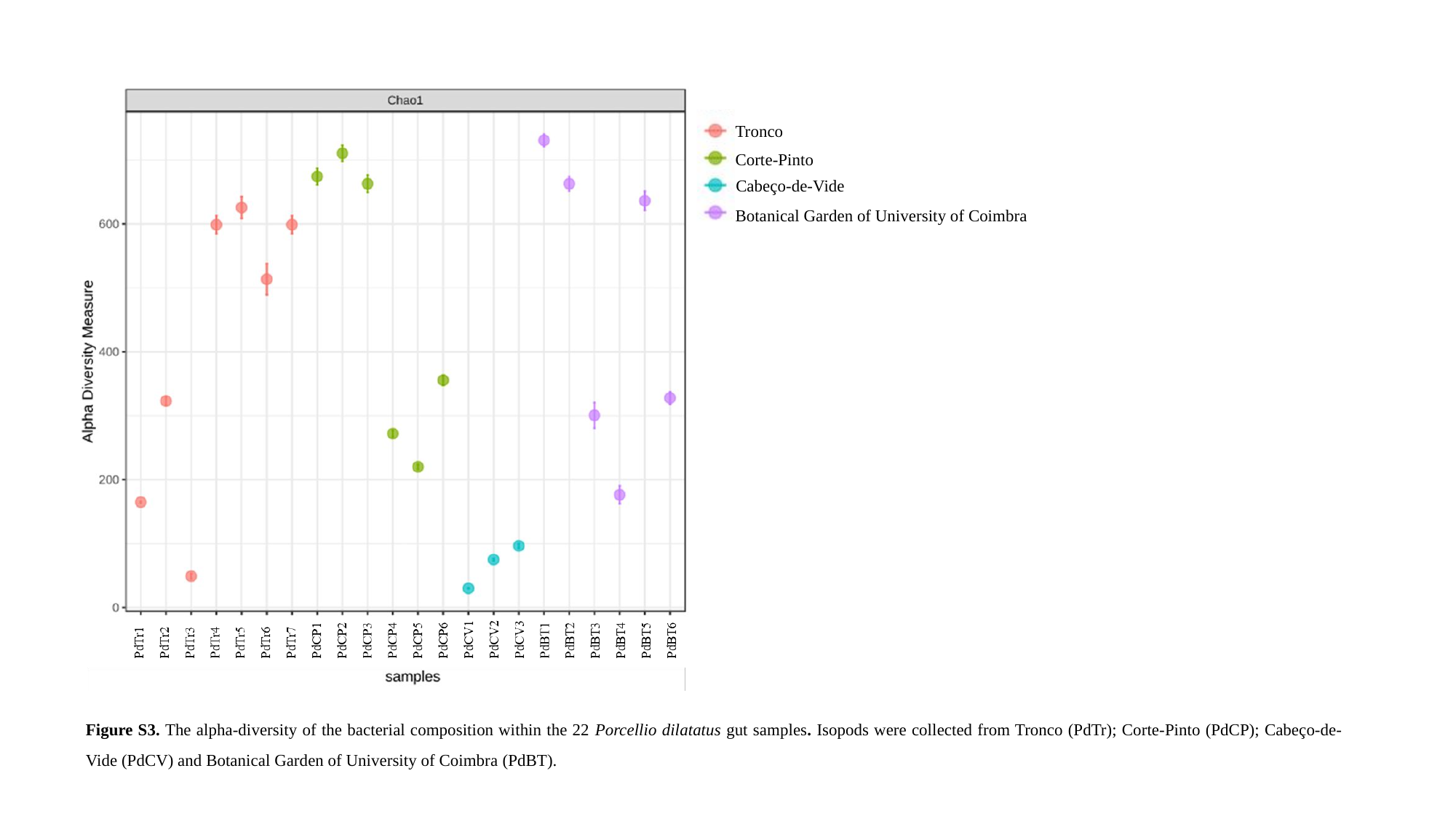

Tronco
Corte-Pinto
Cabeço-de-Vide
Botanical Garden of University of Coimbra
Figure S3. The alpha-diversity of the bacterial composition within the 22 Porcellio dilatatus gut samples. Isopods were collected from Tronco (PdTr); Corte-Pinto (PdCP); Cabeço-de-Vide (PdCV) and Botanical Garden of University of Coimbra (PdBT).
