# Supplementary material for: Guts Bacterial Communities of Porcellio dilatatus: Symbionts Predominance, Functional Significance and Putative Biotechnological Potential
Source: Microorganisms. 2022 Nov 11;10(11):2230. doi: 10.3390/microorganisms10112230 (PMC9692603; doi:10.3390/microorganisms10112230)
Supplement: Supplementary file 1 [file microorganisms-10-02230-s001.zip › TableS1- Alphadiversity indexes_20221103.pdf]

Supplementary tables

Table S1. Alpha-diversity indexes of bacterial communities present in *Porcellio dilatatus* gut samples, calculated by mothur v.1.40.2 software.

Sample ID	Number of sequences	Coverage	Number of OTUs	Inverse simpson	Shannon	Shannon-eveness	Chao
PdTr1	17498	0,998	320	36,5	4,5	0,77	337
PdTr2	12971	0,992	694	14,5	4,8	0,73	767
PdTr3	34836	0,999	160	1,1	0,3	0,07	171
PdTr4	62209	0,994	1488	20,9	4,6	0,63	1738
PdTr5	52998	0,992	1537	32,9	4,8	0,66	1959
PdTr6	53339	0,994	943	2,0	1,8	0,26	1310
PdTr7	61382	0,994	1287	4,2	3,1	0,43	1561
PdCP1	13479	0,995	557	39,3	5,1	0,80	612
PdCP2	12068	0,995	453	29,0	4,7	0,77	495
PdCP3	16144	0,993	679	7,4	3,9	0,60	741
PdCP4	88484	0,995	2000	8,9	3,9	0,51	2377
PdCP5	72436	0,993	2576	40,1	5,5	0,70	2953
PdCP6	62496	0,994	1585	7,4	3,7	0,50	1902
PdCV1	12879	0,998	239	18,0	3,7	0,68	262
PdCV2	28539	1,000	87	10,7	2,8	0,64	88
PdCV3	18477	0,999	251	27,6	4,1	0,75	265
PdBT1	19147	0,997	625	21,6	4,7	0,73	673
PdBT2	16746	0,993	389	14,3	3,4	0,57	480
PdBT3	19294	0,981	1868	76,9	6,2	0,82	2062
PdBT4	13355	0,991	625	26,4	4,5	0,69	702
PdBT5	65918	0,995	3148	212,5	6,7	0,83	3326
PdBT6	73256	0,994	2086	37,7	5,0	0,66	2400
